# Comparison of the Safety and Efficacy of Ustekinumab and Vedolizumab in Patients with Crohn’s Disease: A Systematic Review and Meta-Analysis of Propensity Score Matched Cohort Studies

**DOI:** 10.3390/diseases12110295

**Published:** 2024-11-19

**Authors:** Andrea Pasta, Francesco Calabrese, Elisa Marabotto, Manuele Furnari, Maria Giulia Demarzo, Raffaele Pellegrino, Antonietta Gerarda Gravina, Alessandro Federico, Edoardo Giovanni Giannini, Giorgia Bodini

**Affiliations:** 1 Gastroenterology Unit, Department of Internal Medicine, Istituto di Ricovero e Cura a Carattere Scientifico Policlinico San Martino, University of Genoa, Viale Benedetto XV, 16132 Genoa, Italy; 2 Hepatogastroenterology Division, Department of Precision Medicine, University of Campania Luigi Vanvitelli, Via L. de Crecchio, 80138 Naples, Italy

**Keywords:** Crohn’s Disease, meta-analysis, biologics, ustekinumab, vedolizumab, comparison

## Abstract

**Background**: Ustekinumab and vedolizumab represent both valid therapeutic options in patients with Crohn’s Disease. Data comparing the safety and efficacy of these drugs are indirect, with conflicting results reported. We aim to conduct a systematic review and metanalysis to assess the safety and effectiveness profile of ustekinumab and vedolizumab in patients with Crohn’s Disease, including only studies that applied propensity scores to reduce confounding bias. **Methods**: We identified 59 reports that compared ustekinumab and vedolizumab after a propensity score match analysis, of which 16 were assessed for eligibility, and finally, ten retrospective studies were included. The main outcomes considered were clinical steroid-free remission at 14 ± 4, 24 ± 4, and 52 ± 4 weeks, drug discontinuation rate, adverse events, serious infections, and hospitalization during the first year of treatment. **Results**: A total of 4398 patients were treated with ustekinumab (n = 2774, 63.1%) or vedolizumab (1624, 36.9%). Steroid-free clinical remission was not significantly different between ustekinumab and vedolizumab at 12 ± 4 weeks (OR 1.31, 95%CI 0.88–1.94, *p* = 0.180), at 24 ± 4 weeks (OR 1.18, 95%CI 0.79–1.75, *p* = 0.420), and at 52 ± 4 weeks (1.35, 95%CI 0.91–2.01, *p* = 0.140). In patients receiving ustekinumab, the rate of adverse events (OR 0.54, 95%CI 0.35–0.83, *p* = 0.005), infection (OR 0.61, 95%CI 0.47–0.80, *p* < 0.001) and the need of hospitalization at 1-year (OR 0.68, 95%CI 0.58–0.80, *p* < 0.001) appeared to be lower. **Conclusion**: Ustekinumab and vedolizumab do not significantly differ in inducing and maintaining clinical steroid-free remission, while ustekinumab was associated with a lower risk of serious infections and hospitalization during the first year of treatment.

## 1. Introduction

Crohn’s disease (CD) is a lifelong Inflammatory Bowel Disease characterized by a dysregulation of several immune pathways, leading to an abnormal mucosal immune response and a disrupted epithelial barrier function [1,2]. Alongside the rising incidence of the disease, there has been an increase in innovative therapeutic approaches, which has led to the availability of various treatment options in clinical practice [3,4]. The selection of biological drugs depends on different factors and is driven by local guidelines and economic resources. In Italy, the health care system currently advises the use of an anti-tumor necrosis factor (TNF) drug as a first-line biological agent in patients with CD and steroid-dependent or -refractory disease who are non-responders to conventional therapy [5,6]. Other biological drugs approved as second-line treatment in this scenario are vedolizumab (VDZ), a gut-selective monoclonal antibody directed against α4β7 integrin, and ustekinumab (UST), a human monoclonal antibody directed against the p40 sub-unit of both interleukin-12 and interleukin-23 [7,8]

To the best of our knowledge, there are no studies reporting a direct comparison of second-line biological drugs in patients with CD, and data on safety and efficacy in this scenario are often obtained indirectly [9,10,11,12]. In particular, a direct comparison of the results of different studies is inappropriate due to the heterogeneity of the population included as well as for the possibility of mismatched confounding bias of the population enrolled (e.g., age, comorbidities, disease extension, type, and localization). In order to overcome these limitations, the Propensity Score matching (PSM) can be used to reduce the bias due to confounding variables when evaluating non-head-to-head comparisons. In the absence of direct comparisons, the use of PSM can be regarded as a reliable method to perform a comparison that minimizes the influence of confounding variables, thus allowing for a more accurate evaluation of the relationship between the variables of interest [13]. Recently, several studies used PSM to compare the outcomes of VDZ and UST in patients with CD and moderate-to-severe disease activity, although the results were not consistent. In particular, these studies highlighted variations in response rates, adverse event profiles, and long-term remission rates, shedding light on the need for further investigation into the factors contributing to these discrepancies [8,9,10,11]. So, we felt that a systematic review and a meta-analysis of the published PSM studies in this setting could address this question.

## 2. Methods

### 2.1. Criteria for Study Inclusion

The purpose of the present systematic review with meta-analysis was to compare the safety and effectiveness of VDZ and UST in patients with CD. The Preferred Reporting Items for Systematic Reviews and Meta-Analysis (PRISMA) statement was used to design and conduct the study [14,15].

The studies included in this meta-analysis were those providing comparative data on adverse events (AEs), risk of infection, risk of hospitalization, steroid-free clinical remission (SFR), or risk of drug discontinuation for propensity score-matched CD patients who underwent treatment with UST or VDZ. Propensity score matching was accepted, whereas design and methodology were performed according to the literature recommendation [16,17].

Reviews, editorials, abstracts, and commentaries were excluded. We also excluded studies conducted on populations different from CD (i.e., ulcerative colitis or indeterminate colitis) or on pediatric populations, as well as studies that did not consider our endpoints of interest or where PSM analysis was not comprehensively reported and data on the post-PSM population or pseudopopulation were unavailable.

Treatment with UST or VDZ was defined as the administration of at least one of the scheduled injections according to current guidelines [1,18].

The meta-analysis was registered with the International Platform of Registered Systematic Review and Meta-analysis Protocols (INPLASY), with registration number INPLASY2024110046.

### 2.2. Strategy for Database Search

Two authors independently accessed the online database until 31 December 2023, using PubMed and Ovid, and the research applied search terms referring to “propensity score”, “ustekinumab”, and “vedolizumab”. Supplementary Table S1 reports the detailed web research. Translation of non-English full-text articles was performed with Google Translate, but we were prepared to reach out if additional information or clarifications were required for this meta-analysis. Any disagreement was arbitrated and resolved by discussion with two other authors with extensive experience in IBD management (GB and EGG).

### 2.3. Data Collection and Extraction

The name of the first author, year of publication, total number of patients, and number of patients belonging to UST or VDZ treatment after PSM were collected. Basic patient demographic data (i.e., age, gender, and smoking habits) were also considered, as well as Inflammatory Bowel Disease history (i.e., prior surgery, concomitant medication, extra-intestinal manifestation, and Montreal Classification). The details of the studies are provided in Supplementary Table S2.

Safety analysis was conducted considering the occurrence of AEs following UST or VDZ administration, serious infections in the first year of therapy, defined as infection requiring hospitalization, and the need for hospitalization during the first year of treatment.

Effectiveness was explored by investigating the rate of SFR and drug discontinuation. Clinical remission was considered at 14 ± 4, 24 ± 4, and 52 ± 4 weeks after the first administration of UST or VDZ. The rate of drug discontinuation was collected around the first year (52 ± 4 weeks) of therapy.

### 2.4. Bias Risk Analysis

The risk of bias in non-randomized intervention studies was evaluated using the risk of bias in non-randomized intervention studies (ROBINS I) tool [19]. Two authors (AP and FC) discussed the discrepant results until a consensus was reached. Any disagreements were arbitrated and resolved by discussion with a third author (GB).

The presence of bias due to PSM was assessed according to research methodology checklists in the Propensity Score analysis [16,17], and judgment has been provided in a given section (Supplementary Table S3).

### 2.5. Statistical Analysis and Data Synthesis

Data from individual studies were pooled in a meta-analysis using RevMan 5.4.1 (the Cochrane Collaboration, Copenhagen, Denmark) and ProMeta 3 (IDoStatistics).

Continuous data were synthesized and analyzed using mean and standard deviation (SD). Standard deviations of the mean difference were obtained as previously reported [20]. Whereas the outcome measures were reported in the median and interquartile range (or 95% confidence interval (CI)), mean and SD values were estimated using the previously described methodology [21].

Odds Ratio (OR) was computed using the Mantel-Haenszel OR method [20]. Statistical heterogeneity was reported using I^2^ statistics. The random effect model was used for analysis in the presence of statistical heterogeneity (I^2^ > 50%); otherwise, a fixed effect model was preferred. Two-sided *p* values ≤ 0.05 were considered statistically significant.

Possible publication bias was evaluated with Egger’s regression asymmetry test and Begg’s test, with the results considered to indicate publication bias when *p* < 0.10. If a statistically significant bias was found, the trim and fill method was used for adjustment [22].

## 3. Results

**Figure 1:**
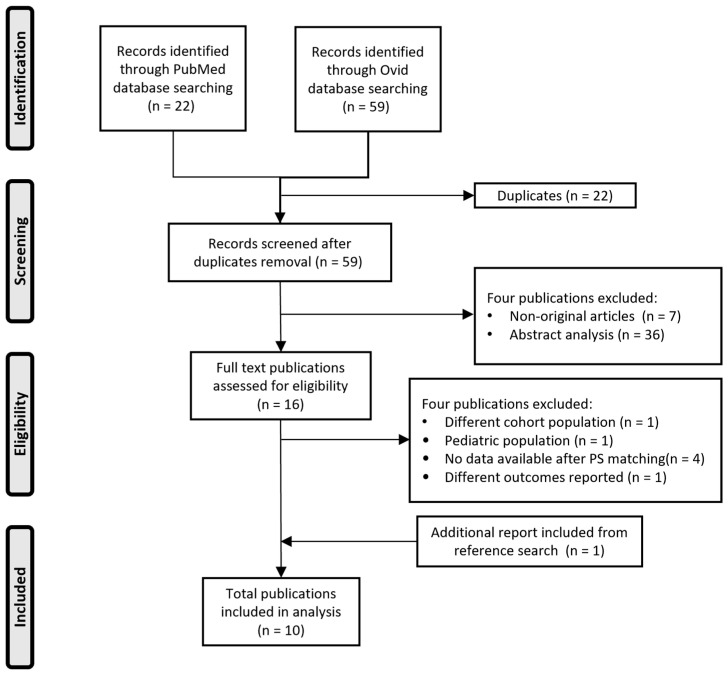
PRISMA flow chart of the literature search and selection process.

Figure 1 presents the detailed sorting process of the publications included in the meta-analysis for a total of ten retrospective and PSM studies [9,11,12,23,24,25,26,27,28,29]. The main characteristics of the studies included in the meta-analysis are reported in Supplementary Table S2. No direct contact with the authors of the included studies was required for this meta-analysis. Supplementary Table S3 reports the PRISMA 2020 checklist.

A total of 4398 patients with CD were included in the analysis. Their mean age was 42.1 ± 14.6 years, and there was a slight prevalence of female gender at 52.2% (n = 2294/4398), while the proportion of actively smoking patients ranged between 10% and 38% (n = 608/2588), 23.5%, available in 8/10 studies.

The most prevalent disease behavior was non-structuring and non-penetrating (n = 1138/2567, 44.3%, available in 7/10 studies), while ileocolic localization was prevalent (n = 1025/2567, 39.9%, available in 7/10 studies). Disease duration was 11.8 ± 9.1 years (available in 2089 patients, 6/10 studies), and patients who underwent bowel resection were 28.1% (n = 1064/3784, available in 8/10 studies).

Overall, 2774 (63.1%) subjects were treated with UST, while 1624 (36.9%) patients were treated with VDZ. The rates of concomitant systemic steroid and immunomodulator administrations at baseline were 1248/3956 (31.5%) and 843/3956 (28.5%) in patients treated with UST or with VDZ, respectively (available in 9/10 studies). The general characteristics of patients for each study are summarised in Table 1.

### 3.1. Quality of Included Studies

**Figure 2:**
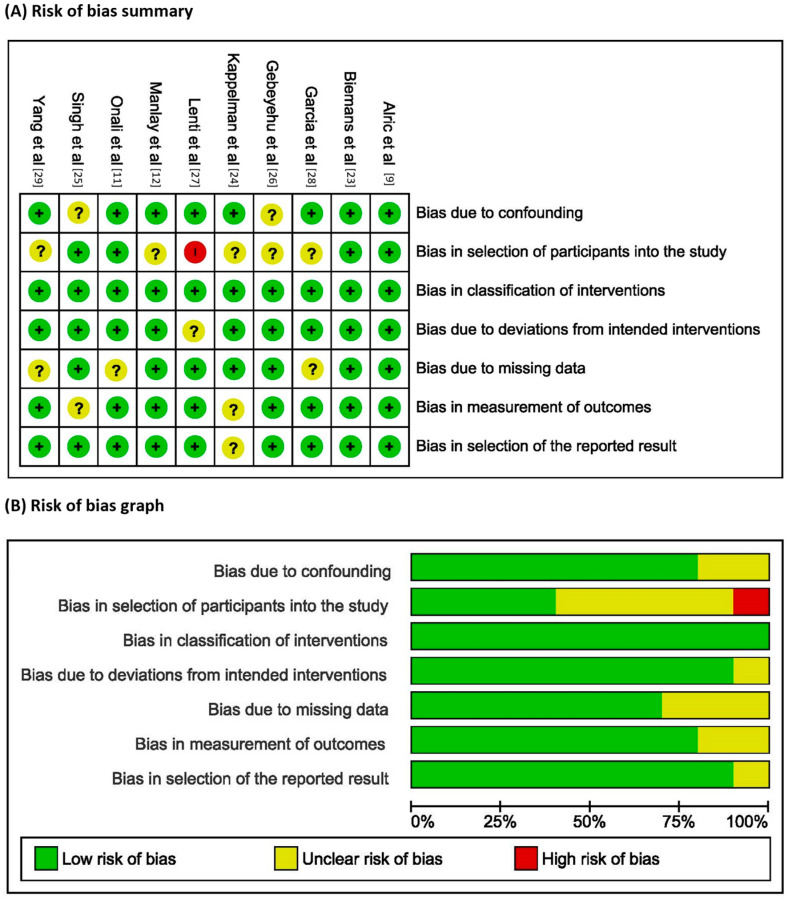
(**A**) Risk of bias summary: review of authors’ judgment about each risk of bias item for each included study, and (**B**) Risk of bias graph: review of authors’ judgment about each risk of bias item presented as percentages across all included studies.

The detailed bias analysis is shown in Supplementary Table S4. None of the included studies was judged to be at critical risk of bias, but only two studies received a low risk of bias judgment. Seven out of ten studies were considered at moderate risk of bias, while one study received a high risk of bias judgment. Figure 2A shows the risk of bias summary, which reports the authors’ judgment about each risk of bias item for each included study, while the risk of bias graph, which reports review authors’ judgments about each risk of bias item across all included studies, is shown in Figure 2B.

### 3.2. Effectiveness

The rate of 52-week SFR was reported in eight studies, although 12-week and 24-week SFR was available only in five and six studies, respectively.

**Figure 3:**
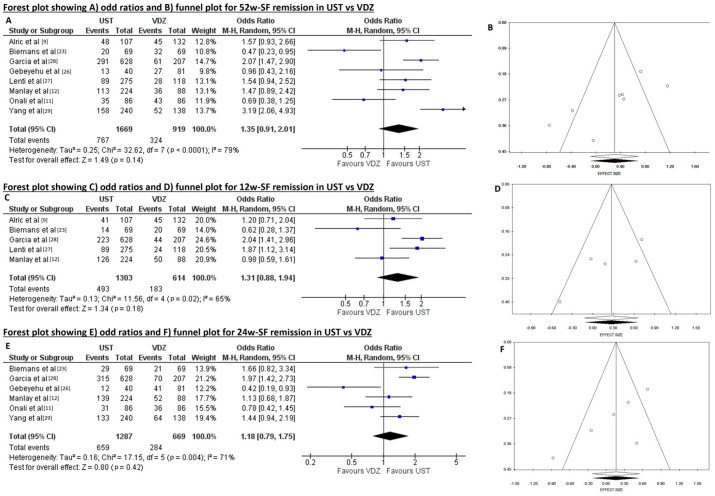
(**A**) Forest plot showing odd ratios and (**B**) funnel plot for 52 weeks (w) steroid-free remission (SF). (**C**) Forest plot showing odds ratios and (**D**) funnel plot for 12w-SF remission. (**E**) Forest plot showing odds ratios and (**F**) funnel plot for 24 w-SF remission in ustekinumab (UST) and vedolizumab (VDZ).

A total of 1091/2588 (42.2%) patients experienced SFR at 52 weeks, resulting in 767/1669 (46.0%) with UST versus 324/919 (35.3%) with VDZ therapy. When data were pooled, there was high heterogeneity (Tau^2^ = 0.25, *p* < 0.001, I^2^ = 79%), and the pooled OR of 52-week SFR was not significantly different between UST and VDZ (1.35, 95CI 0.91–2.01, *p* = 0.14). The risk of publication bias using Begg’s (*p* = 0.021) and Egger’s (*p* = 0.027) tests was high, but the funnel plot appeared symmetrical, and no study was trimmed at the trim and fill test (Figure 3A,B).

The 12-week SFR was achieved in 676/1917 patients (35.3%), of which 493/1303 (37.8%) treated with UST and 183/614 (29.8%) treated with VDZ. When data were pooled, there was high heterogeneity (Tau^2^ = 0.13, *p* = 0.02, I^2^ = 65%), and there was no significant difference between UST and VDZ (OR 1.31, 95CI 0.88–1.94, *p* = 0.180). Begg’s (*p* = 0.142) and Egger’s (*p* = 0.087) tests showed no risk of publication bias, but the funnel plot was symmetrical, and no study was trimmed at the trim and fill test (Figure 3C,D).

Likewise, the 24-week SFR was obtained in 943/1956 (48.2%) patients, meaning 659/1287 (51.2%) treated with UST and 284/669 (42.5%) treated with VDZ. The heterogeneity was high in pooled analysis (Tau^2^ = 0.16, *p* = 0.004, I^2^ = 71%), and there was no significant difference between UST and VDZ (OR 1.18, 95CI 0.79–1.75, *p* = 0.420). The risk of publication bias using Begg’s (*p* = 0.091) and Egger’s (*p* = 0.052) tests was low, and the funnel plot appeared symmetrical, as confirmed by the trim and fill test (Figure 3E,F).

**Figure 4:**
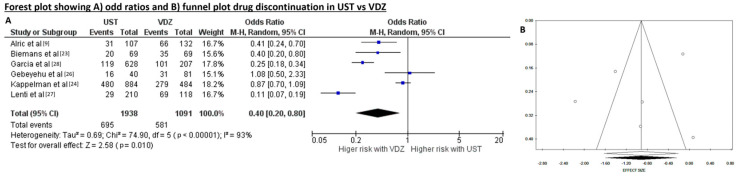
(**A**) Forest plot showing odds ratios and (**B**) funnel plot for 1-year drug discontinuation in ustekinumab (UST) and vedolizumab (VDZ).

The rate of drug discontinuation was reported in six studies for a total of 3029 patients, with a lower risk in patients treated with UST (n = 695/1938, 35.9%) than in those who received VDZ (n = 581/1091, 53.3%) in the non-weighted baseline analysis. When data were pooled, there was high heterogeneity (Tau^2^ = 0.69, *p* = 0 < 0.001, I^2^ = 93%), but the risk of publication bias using Begg’s (*p* = 0.573) and Egger’s (*p* = 0.378) tests was low, and the funnel plot appeared symmetrical as confirmed by trim and fill test. The OR of drug discontinuation was 0.40 (95IC 0.20–0.80, *p* = 0.010) in the UST and VDZ comparison, meaning a significant difference of higher drug discontinuation risk in patients receiving VDZ (Figure 4A,B).

### 3.3. Safety: Risk of Adverse Events, Hospitalization, and Infection

**Figure 5:**
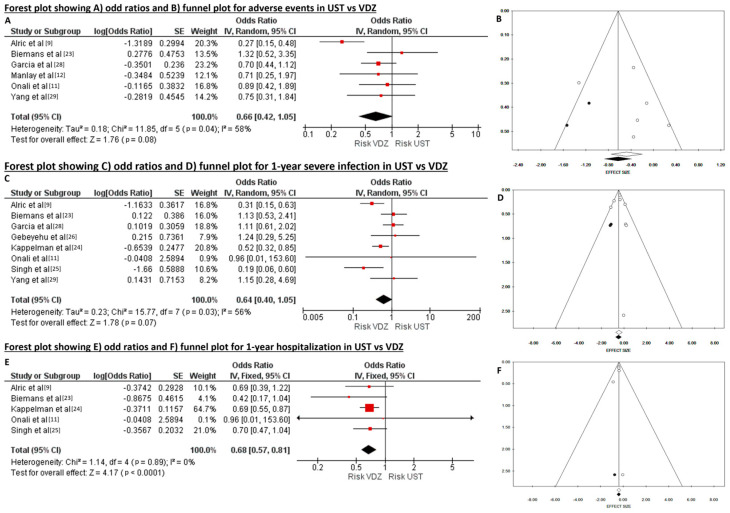
(**A**) Forest plot showing odd ratios and (**B**) funnel plot for adverse events. (**C**) Forest plot showing odds ratios and (**D**) funnel plot for 1-year severe infection. (**E**) Forest plot showing odds ratios and (**F**) funnel plot for 1-year hospitalization in ustekinumab (UST) and vedolizumab (VDZ).

Overall, six studies reported the occurrence of AEs during the administration of UST or VDZ for a total of 2074 patients. When data were pooled, there was evidence of heterogeneity (Tau^2^ = 0.18, *p* = 0.04, I^2^ = 58%), and no publication bias was found using Begg’s (*p* = 0.851) and Egger’s (*p* = 0.469) tests. No significant difference in the risk of AEs between UST and VDZ was identified (OR 0.66, CI95 0.42–1.05, *p* = 0.080). The funnel plot was asymmetrical, as revealed in trim and fill analysis, which determined the trimming of two studies. The adjusted OR improved and reached statistical significance (OR 0.54, 95IC 0.35–0.83, *p* = 0.005) (Figure 5A,B).

The rate of infection at 1-year was investigated in eight studies involving a total of 3693 patients treated with UST (n = 2275, 61.6%) or VDZ (n = 1418, 38.4%). The pooled OR was 0.64 (CI95 0.40–1.05, *p* = 0.070), showing a lower trend of 1-year risk of infection in patients receiving UST. The heterogeneity was borderline high (Tau^2^ = 0.23, *p* = 0.03, I^2^ = 56%), and no publication bias was found using Begg’s (*p* = 0.862) and Egger’s (*p* = 1.000) tests. Due to the asymmetrical appearance of the funnel plot, the ‘trim and fill’ method was applied, and two studies were trimmed. The re-estimated OR slightly decreased and became significantly different between the two groups (OR 0.61, 95CI 0.47–0.80, *p* < 0.001) (Figure 5C,D).

Five studies reported the need for hospitalization after 1-year of treatment with either UST or VDZ, including a total of 2359 patients (UST, n = 1367, 57.9%; VDZ, n = 992, 42.1%). When data were pooled, there was no evidence of heterogeneity (Chi^2^ = 1.14, *p* = 0.89, I^2^ = 0%), and the pooled OR was 0.68 (CI95 0.57–0.81, *p* < 0.0001) for UST compared to VDZ treatment. The risk of publication bias using Begg’s (*p* = 0.327) and Egger’s (*p* = 0.455) tests was low, but the funnel plot appeared asymmetrical, and the ‘trim and fill’ method was applied, trimming one study. The re-estimated odds ratio (OR) maintained its statistical significance, indicating that patients treated with UST have a lower risk of experiencing hospitalization within one year compared to those treated with VDZ (OR 0.68, CI95 0.58–0.80, *p* < 0.0001) (Figure 5E,F).

## 4. Discussion

The CD is a chronic relapsing inflammatory bowel disease that has a great impact on a patient’s quality of life [30]. The proportion of patients with CD non-responders or who face secondary loss of response to anti-TNF drugs is consistent [31,32]. To date, two second-line treatments are available, with different mechanisms of action, VDZ and UST, an anti-integrin and an anti-interleukin, respectively. Head-to-head randomized clinical trials comparing VDZ and UST are not available, and therefore, clinical decisions in the management of patients with CD who need a second-line biological drug after anti-TNF are nowadays based both on physician personal experience and on the results of the few comparative studies available [9,10,12,33].

In this systematic review and meta-analysis, we sought to compare the reported safety and effectiveness of UST and VDZ in PSM studies in order to address this question on this relevant issue. To our knowledge, this is the first systematic review and meta-analysis specifically investigating data from PSM studies. Indeed, Parrott et al. previously conducted a meta-analysis comparing the efficacy profiles of VDZ and UST in patients with CD refractory to anti-TNF drugs, concluding that UST appears to be more effective than VDZ in maintenance of remission, although this analysis included five observational studies in the final analysis and did not apply any strategy to avoid confounders [33]. In order to overcome these limitations and to provide unbiased results, in the present meta-analysis, we considered only manuscripts that used PSM to adjust for high-dimensional confounders so as to provide a more precise evaluation of the comparison proposed [34].

In this meta-analysis, UST and VDZ seem to be equally effective in inducing 12-week clinical remission as well as in maintaining SFR both at 24- and 52-week. In real-world practice, failure after first-line therapy with anti-TNF is a common occurrence and ranges between 13% and 20% [31,32]. Our results may support clinicians who consider second-line treatment following anti-TNF failure, as both UST and VDZ represent valid therapeutic options to obtain and maintain remission in patients with moderate to severe activity of disease [35,36,37]. Indeed, given the similar rate of efficacy, the choice between these two drugs can be balanced on the patient’s preference or on the administration route as induction is administered intravenously for both drugs, while UST has a subcutaneous maintenance schedule, while VDZ was originally developed for intravenous maintenance and only recently was approved for subcutaneous use [5,6,38].

In our meta-analysis, patients treated with VDZ had a higher risk of drug discontinuation before 12 months of treatment (*p* = 0.010). While some studies used pharmacy registries to report the reason for drug withdrawal, in this meta-analysis, the manuscripts included indicated only the number of patients who discontinued treatment during the 12-month follow-up. Therefore, the reasons leading to drug discontinuation in these populations could be only speculated: drug failure, the occurrence of AEs, disease remission, physicians’ choice, or patients’ preference. Likewise, its impact on response could not be assessed.

Another pivotal point that guides the choice of physicians for the treatment of patients with CD is the safety profile of the proposed therapies. According to the results of our meta-analysis, in PSM patients, we observed a statistically significant difference in the rate of overall AEs in favor of UST, as well as treatment with UST seemed to have a better safety profile in terms of serious infections and hospitalization rates. Unfortunately, our findings are driven primarily by four studies [9,24,25,28], and among these, one study alone included the majority of patients considered to evaluate this issue. Moreover, none of these studies exhaustively reported data on steroid therapy administered after baseline, and the unknown amount of steroid administered during the 52 weeks of treatment with UST or VDZ may explain the different rates of infection that emerged from this analysis [9,24,25,28]. In fact, the safety profile of a specific drug in CD depends on three main factors. Firstly, the intrinsic immunosuppressive effect of the agent should be considered, followed by the power to control disease activity, and lastly, the efficacy in avoiding concomitant steroid administration. From a theoretical point of view, VDZ, being a gut-selective molecule, is supposed to be safer than UST, which is an anti-interleukin antibody with a potential systemic immunosuppressive action. Thus, without solid information on the use of steroid therapies among patients in these studies, it is challenging to make a definitive assessment. Steroids, often used alongside these biological therapies to manage flare-ups in CD, are known to carry significant risks of infection, hospitalization, and other adverse effects [39]. Their use could skew the safety profiles of both drugs by either amplifying or diminishing their apparent risks and efficacy, which may have affected both the efficacy and the risk of infection and, as a result, hospitalization. Moreover, while the clinical response to VDZ compared to placebo is reported to be significantly different by week six, the peak effect may not occur until weeks 10–14, which is generally longer than for other biologic drugs [40]. This delay may have led to a more frequent early use of corticosteroids. Among patients receiving corticosteroids or a combination of corticosteroids and immunomodulators at baseline, a higher proportion of those treated with VDZ achieved clinical remission and showed an improved clinical response by week six [41].

The main strength of our analysis is the inclusion of only manuscripts that used PSM. While it is well known that the use of PSM leads to an intrinsic reduction of the sample size of the population under study due to its statistical significance, the numerosity of the population we analyzed was consistent.

This study has undoubted limitations. First, the design of all studies included in the meta-analysis was retrospective, and most of the studies were evaluated by authors as being at moderate risk of bias (although no study was considered at high risk of bias). The retrospective nature of these studies inherently restricts the ability to draw firm conclusions regarding causality, which can introduce potential inaccuracies and biases. Retrospective studies are also generally less robust than prospective studies in controlling for confounding factors. Second, the application of PSM may have determined imbalance because of incomplete matching, especially in the studies in which the matched sample size was less than 50%. Moreover, another limitation is the lack of data regarding the intensification of treatment by reducing the administration intervals between two consecutive therapies based on clinical judgment and without a pre-defined parameter. Lastly, the median follow-up time in the studies that evaluate UST and VDZ in this setting is no longer than 12 months.

## 5. Conclusions

To conclude, both UST and VDZ are effective options for patients with CD, although UST seems to be associated with a lower risk of infections and hospitalization and overall AEs. As the majority of studies had a relevant bias, we feel that a definite answer regarding the comparison of UST and VDZ in patients with CD may be provided only by prospective, randomized, comparative studies so as to better guide the clinician in the choice of the second-line treatment in these patients.

## Figures and Tables

**Table 1:** Baseline demographic and clinical features among patients included in the studies analyzed.

	Biemans et al. 2020 [23] n = 138	Gebeyehu et al. 2022 [26] n = 121	Kappelman et al. 2022 [24] n = 1368	Lenti et al. 2021 [27] n = 393	Manlay et al. 2021 [12] n = 312	Onali et al. 2022 [11] n = 172	Singh et al. 2022 [25] n = 442	Alric et al. 2020 [9] n = 239	Yang et al. 2023 [29] n = 378	Garcia et al. 2024 [28] n = 835
**Gender, male [n** (**%**)**]**	47 (34.0)	44 (36.4)	597 (43.6)	140 (35.6)	198 (63.5)	93 (54.3)	184 (83.3)	109 (45.6)	251 (66.4)	420 (50.3)
**Age [years]**	37.4 ± 19	68 ± 6.4	42.4 ±14.5	41 ± 15	38.5 ± 13.2	43 ± 16	40 ± 17.4	39.5 ± 14.5	34.3 ± 11.8	45.6 ± 15.3
**Active smoke [n** (**%**)**]**	32 (25)	15 (12.0)		79 (20.1)	97 (31.2)	65 (37.8)		76 (31.8)	39 (10.3)	205 (24.5)
**Age at diagnosis [years]**						29 ± 12.8				33.2 ± 16
**Disease duration [years]**	12.2 ± 8.9			11 ± 9	11.8 ± 9.1	11 ± 8		11.3 ± 7.8		12.5 ± 9.6
**Prior surgery [n** (**%**)**]**	84 (60.1)	56 (46.3)	109 (8.0)	98 (25.0)	114 (36.4)			99 (41.5)	125 (33.1)	379 (45.4)
**Concomitant medication [n** (**%**)**]**										
*Steroids at baseline*	24 (17.4)	40 (33.0)	369 (27.0)	141 (35.8)	263 (84.3)	73 (42.4)		97 (40.6)	25 (6.6)	216 (25.9)
*Immunosuppressors at baseline*	32 (23.2)	15 (12.4)	175 (12.8)	157 (39.9)	87 (27.8)	17 (9.9)		82 (34.3)	5 (1.3)	273 (32.7)
**Montreal behaviour [n** (**%**)**]**										
*B1: non-stricturing, non-penetrating*	66 (47.8)			138 (35.1)	135 (43.2)	63 (36.6)		143 (59.8)	170 (45.0)	424 (50.8)
*B2: stricturing*	41 (29.7)			142 (36.2)	177 (56.7)	74 (43.0)		38 (15.9)	95 (25.1)	201 (24.1)
*B3: penetrating*	29 (21.0)	18 (14.8)		113 (28.7)		31 (18.0)		58 (24.3)	108 (28.6)	210 (25.1)
**Montreal localization [n** (**%**)**]**										
*L1: terminal ileal*	44 (31.8)			59 (15.0)	249 (79.8) ^1^	54 (31.4)		80 (33.5)	58 (15.3)	380 (45.5)
*L2: colonic*	42 (30.4)			212 (54.0)	63 (20.3)	17 (9.9)		50 (20.9)	10 (2.6)	105 (12.6)
*L3: ileocolic*	42 (30.4)			122 (31.0)		97 (56.4)		104 (43.5)	310 (82.0)	350 (41.9)
*L4: isolated upper GI*	10 (7.0)					11 (6.4)		5 (2.4)	21 (5.5)	87 (10.4)
**Perianal disease [n** (**%**)**]**	20 (14.4)	10 (0.8)	127 (9.0)		150 (48.1)			118 (49.4)	197 (52.1)	284 (34.0)

Continuous data are shown as median and IQR, and nominal data as number (% patients). ^1^ This study reported L1 + L3 in the same group.

## Data Availability

No new data were created or analyzed in this study. Data sharing is not applicable to this article.

## Supplementary Materials

The following supporting information can be downloaded at: https://www.mdpi.com/article/10.3390/diseases12110295/s1, Table S1: Search strategy of electronic databases; Table S2: Studies included in meta-analysis; Table S3: PRISMA 2020 checklist; Table S4: Detailed bias risks report with authors’ statements.

## References

[1] Gomollón F., Dignass A., Annese V., Tilg H., Van Assche G., Lindsay J.O., Peyrin-Biroulet L., Cullen G.J., Daperno M., Kucharzik T. (2017). 3rd European Evidence-Based Consensus on the Diagnosis and Management of Crohn’s Disease 2016: Part 1: Diagnosis and Medical Management. J. Crohns Colitis.

[2] Torres J., Mehandru S., Colombel J.-F., Peyrin-Biroulet L. (2017). Crohn’s Disease. Lancet.

[3] Gubatan J., Keyashian K., Rubin S.J.S., Wang J., Buckman C.A., Sinha S. (2021). Anti-Integrins for the Treatment of Inflammatory Bowel Disease: Current Evidence and Perspectives. Clin. Exp. Gastroenterol..

[4] Macaluso F.S., Orlando A., Cottone M. (2019). Anti-Interleukin-12 and Anti-Interleukin-23 Agents in Crohn’s Disease. Expert Opin. Biol. Ther..

[5] Gazzetta Ufficiale. https://www.gazzettaufficiale.it/atto/serie_generale/caricaDettaglioAtto/originario?atto.dataPubblicazioneGazzetta=2021-07-22&atto.codiceRedazionale=21A04297&elenco30giorni=true.

[6] Gazzetta Ufficiale. https://www.gazzettaufficiale.it/atto/serie_generale/caricaDettaglioAtto/originario?atto.dataPubblicazioneGazzetta=2022-05-21&atto.codiceRedazionale=22A02953&elenco30giorni=true.

[7] Sandborn W.J., Feagan B.G., Rutgeerts P., Hanauer S., Colombel J.-F., Sands B.E., Lukas M., Fedorak R.N., Lee S., Bressler B. (2013). Vedolizumab as Induction and Maintenance Therapy for Crohn’s Disease. N. Engl. J. Med..

[8] Sandborn W.J., Feagan B.G., Danese S., O’Brien C.D., Ott E., Marano C., Baker T., Zhou Y., Volger S., Tikhonov I. (2020). Safety of Ustekinumab in Inflammatory Bowel Disease: Pooled Safety Analysis of Results from Phase 2/3 Studies. Inflamm. Bowel Dis..

[9] Alric H., Amiot A., Kirchgesner J., Tréton X., Allez M., Bouhnik Y., Beaugerie L., Carbonnel F., Meyer A. (2020). The Effectiveness of Either Ustekinumab or Vedolizumab in 239 Patients with Crohn’s Disease Refractory to Anti-Tumour Necrosis Factor. Aliment. Pharmacol. Ther..

[10] Townsend T., Razanskaite V., Dodd S., Storey D., Michail S., Morgan J., Davies M., Penman D., Watters C., Swaminathan M. (2020). Comparative Effectiveness of Ustekinumab or Vedolizumab after One Year in 130 Patients with Anti-TNF-Refractory Crohn’s Disease. Aliment. Pharmacol. Ther..

[11] Onali S., Pugliese D., Caprioli F.A., Orlando A., Biancone L., Nardone O.M., Imperatore N., Fiorino G., Cappello M., Viola A. (2022). An Objective Comparison of Vedolizumab and Ustekinumab Effectiveness in Crohn’s Disease Patients’ Failure to TNF-Alpha Inhibitors. Am. J. Gastroenterol..

[12] Manlay L., Boschetti G., Pereira B., Flourié B., Dapoigny M., Reymond M., Sollelis E., Gay C., Boube M., Buisson A. (2021). Comparison of Short- and Long-Term Effectiveness between Ustekinumab and Vedolizumab in Patients with Crohn’s Disease Refractory to Anti-Tumour Necrosis Factor Therapy. Aliment. Pharmacol. Ther..

[13] Rosenbaum P.R., Rubin D.B. (1983). The Central Role of the Propensity Score in Observational Studies for Causal Effects. Biometrika.

[14] Liberati A., Altman D.G., Tetzlaff J., Mulrow C., Gøtzsche P.C., Ioannidis J.P.A., Clarke M., Devereaux P.J., Kleijnen J., Moher D. (2009). The PRISMA Statement for Reporting Systematic Reviews and Meta-Analyses of Studies That Evaluate Healthcare Interventions: Explanation and Elaboration. BMJ.

[15] Page M.J., McKenzie J.E., Bossuyt P.M., Boutron I., Hoffmann T.C., Mulrow C.D., Shamseer L., Tetzlaff J.M., Akl E.A., Brennan S.E. (2021). The PRISMA 2020 Statement: An Updated Guideline for Reporting Systematic Reviews. BMJ.

[16] Rb D. (1998). Propensity Score Methods for Bias Reduction in the Comparison of a Treatment to a Non-Randomized Control Group. Stat. Med..

[17] Yao X.I., Wang X., Speicher P.J., Hwang E.S., Cheng P., Harpole D.H., Berry M.F., Schrag D., Pang H.H. (2017). Reporting and Guidelines in Propensity Score Analysis: A Systematic Review of Cancer and Cancer Surgical Studies. J. Natl. Cancer Inst..

[18] Macaluso F.S., Papi C., Orlando A., Festa S., Pugliese D., Bonovas S., Pansieri C., Piovani D., Fiorino G., Fantini M.C. (2023). Use of Biologics for the Management of Crohn’s Disease: IG-IBD Clinical Guidelines Based on the GRADE Methodology. Dig. Liver Dis..

[19] Sterne J.A., Hernán M.A., Reeves B.C., Savović J., Berkman N.D., Viswanathan M., Henry D., Altman D.G., Ansari M.T., Boutron I. (2016). ROBINS-I: A Tool for Assessing Risk of Bias in Non-Randomised Studies of Interventions. BMJ.

[20] Follmann D., Elliott P., Suh I., Cutler J. (1992). Variance Imputation for Overviews of Clinical Trials with Continuous Response. J. Clin. Epidemiol..

[21] Wan X., Wang W., Liu J., Tong T. (2014). Estimating the Sample Mean and Standard Deviation from the Sample Size, Median, Range and/or Interquartile Range. BMC Med. Res. Methodol..

[22] Lin L., Chu H. (2018). Quantifying Publication Bias in Meta-Analysis. Biometrics.

[23] Biemans V.B.C., van der Woude C.J., Dijkstra G., van der Meulen-de Jong A.E., Löwenberg M., de Boer N.K., Oldenburg B., Srivastava N., Jansen J.M., Bodelier A.G.L. (2020). Ustekinumab Is Associated with Superior Effectiveness Outcomes Compared to Vedolizumab in Crohn’s Disease Patients with Prior Failure to Anti-TNF Treatment. Aliment. Pharmacol. Ther..

[24] Kappelman M.D., Adimadhyam S., Hou L., Wolfe A.E., Smith S., Simon A.L., Moyneur É., Reynolds J.S., Toh S., Dobes A. (2022). Real-World Evidence Comparing Vedolizumab and Ustekinumab in Antitumor Necrosis Factor-Experienced Patients With Crohn’s Disease. Am. J. Gastroenterol..

[25] Singh S., Kim J., Luo J., Paul P., Rudrapatna V., Park S., Zheng K., Syal G., Ha C., Fleshner P. (2022). Comparative Safety and Effectiveness of Biologic Therapy for Crohn’s Disease: A CA-IBD Cohort Study. Clin. Gastroenterol. Hepatol..

[26] Gebeyehu G.G., Fiske J., Liu E., Limdi J.K., Broglio G., Selinger C., Razsanskaite V., Smith P.J., Flanagan P.K., Subramanian S. (2022). Ustekinumab and Vedolizumab Are Equally Safe and Effective in Elderly Crohn’s Disease Patients. Dig. Dis. Sci..

[27] Lenti M.V., Dolby V., Clark T., Hall V., Tattersall S., Fairhurst F., Kenneth C., Walker R., Kemp K., Borg-Bartolo S. (2022). A Propensity Score-Matched, Real-World Comparison of Ustekinumab vs Vedolizumab as a Second-Line Treatment for Crohn’s Disease. The Cross Pennine Study II. Aliment. Pharmacol. Ther..

[28] García M.J., Rivero M., Fernández-Clotet A., de Francisco R., Sicilia B., Mesonero F., de Castro M.L., Casanova M.J., Bertoletti F., García-Alonso F.J. (2024). Comparative Study of the Effectiveness of Vedolizumab Versus Ustekinumab After Anti-TNF Failure in Crohn’s Disease (Versus-CD): Data from the ENEIDA Registry. J. Crohns Colitis.

[29] Yang H., Huang Z., Li M., Zhang H., Fu L., Wang X., Yang Q., He Y., Wu W., Jiang T. (2023). Comparative Effectiveness of Ustekinumab vs. Vedolizumab for Anti-TNF-Naïve or Anti-TNF-Exposed Crohn’s Disease: A Multicenter Cohort Study. EClinicalMedicine.

[30] Knowles S.R., Graff L.A., Wilding H., Hewitt C., Keefer L., Mikocka-Walus A. (2018). Quality of Life in Inflammatory Bowel Disease: A Systematic Review and Meta-Analyses-Part I. Inflamm. Bowel Dis..

[31] Billioud V., Sandborn W.J., Peyrin-Biroulet L. (2011). Loss of Response and Need for Adalimumab Dose Intensification in Crohn’s Disease: A Systematic Review. Am. J. Gastroenterol..

[32] Gisbert J.P., Panés J. (2009). Loss of Response and Requirement of Infliximab Dose Intensification in Crohn’s Disease: A Review. Am. J. Gastroenterol..

[33] Parrot L., Dong C., Carbonnel F., Meyer A. (2022). Systematic Review with Meta-Analysis: The Effectiveness of Either Ustekinumab or Vedolizumab in Patients with Crohn’s Disease Refractory to Anti-Tumour Necrosis Factor. Aliment. Pharmacol. Ther..

[34] Vansteelandt S., Daniel R.M. (2014). On Regression Adjustment for the Propensity Score. Stat. Med..

[35] Feagan B.G., Rutgeerts P., Sands B.E., Hanauer S., Colombel J.-F., Sandborn W.J., Van Assche G., Axler J., Kim H.-J., Danese S. (2013). Vedolizumab as Induction and Maintenance Therapy for Ulcerative Colitis. N. Engl. J. Med..

[36] Vermeire S., Loftus E.V., Colombel J.-F., Feagan B.G., Sandborn W.J., Sands B.E., Danese S., D’Haens G.R., Kaser A., Panaccione R. (2017). Long-Term Efficacy of Vedolizumab for Crohn’s Disease. J. Crohns Colitis.

[37] Feagan B.G., Sandborn W.J., Gasink C., Jacobstein D., Lang Y., Friedman J.R., Blank M.A., Johanns J., Gao L.-L., Miao Y. (2016). Ustekinumab as Induction and Maintenance Therapy for Crohn’s Disease. N. Engl. J. Med..

[38] Vermeire S., D’Haens G., Baert F., Danese S., Kobayashi T., Loftus E.V., Bhatia S., Agboton C., Rosario M., Chen C. (2021). Efficacy and Safety of Subcutaneous Vedolizumab in Patients with Moderately to Severely Active Crohn’s Disease: Results From the VISIBLE 2 Randomised Trial. J. Crohns Colitis.

[39] Farraj K.L., Pellegrini J.R., Munshi R.F., Russe-Russe J., Kaliounji A., Tiwana M.S., Srivastava P., Subramani K. (2022). Chronic Steroid Use: An Overlooked Impact on Patients with Inflammatory Bowel Disease. JGH Open.

[40] Shim H.H., Chan P.W., Chuah S.W., Schwender B.J., Kong S.C., Ling K.L. (2018). A Review of Vedolizumab and Ustekinumab for the Treatment of Inflammatory Bowel Diseases. JGH Open.

[41] Colombel J.-F., Loftus E.V., Siegel C.A., Lewis J.D., Smyth M., Xu J., Abhyankar B. (2015). P528. Efficacy of Vedolizumab with Concomitant Corticosteroid or Immunomodulator Use in Patients with Crohn’s Disease in GEMINI 2. J. Crohn’s Colitis.

