# Supplementary material for: Comparison of the Safety and Efficacy of Ustekinumab and Vedolizumab in Patients with Crohn’s Disease: A Systematic Review and Meta-Analysis of Propensity Score Matched Cohort Studies
Source: Diseases. 2024 Nov 19;12(11):295. doi: 10.3390/diseases12110295 (PMC11593252; doi:10.3390/diseases12110295)
Supplement: Supplementary file 1 [file diseases-12-00295-s001.zip › diseases-3277167-supplementary.pdf]

Summary

Supplementary Table S1. Search strategy of electronic databases 2

Supplementary Table S2. Studies included in meta-analysis 3

Supplementary Table S3. PRISMA 2020 check list. 4

Supplementary Table S4. Detailed bias risks report with authors' statements 9

Supplementary Table S1. Search strategy of electronic databases

Search Ovid Table of Contents and Abstracts
EBM Reviews - ACP Journal Club <1991 to December 2023, 31st >
EBM Reviews - Cochrane Central Register of Controlled Trials < December 2023, 31st >
EBM Reviews - Cochrane Database of Systematic Reviews <2005 to December 2023, 31th >
EBM Reviews - Cochrane Clinical Answers < December 2023, 31th >
EBM Reviews - Database of Abstracts of Reviews of Effects <1st Quarter 2016>
EBM Reviews - Cochrane Methodology Register <3rd Quarter 2012>
EBM Reviews - Health Technology Assessment <4th Quarter 2016>
EBM Reviews - NHS Economic Evaluation Database <1st Quarter 2016>
Ovid Healthstar <1966 to December 2023, 31th >
Ovid MEDLINE(R) ALL <1946 to December 2023, 31th >
APA PsycInfo <1806 to December 2023, 31th >
Daily and Versions < access the online database until December 2022, 31th>

1. Propensity score.mp. [mp=tx, bt, ti, ot, ab, ct, sh, kw, fx, hw, nm, kf, ox, px, rx, an, ui, ds, on, sy, ux, mx, tc, id, tm, mf] : n= 120869
2. Limit 1 to abstracts: n= 102086
3. Vedolizumab.mp. [mp=tx, bt, ti, ot, ab, ct, sh, kw, fx, hw, nm, kf, ox, px, rx, an, ui, ds, on, sy, ux, mx, tc, id, tm, mf]: n= 7214
4. Limit 3 to abstracts: n= 4443
5. Ustekinumab.mp. [mp=tx, bt, ti, ot, ab, ct, sh, kw, fx, hw, nm, kf, ox, px, rx, an, ui, ds, on, sy, ux, mx, tc, id, tm, mf]: n= 12769
6. Limit 5 to abstracts: n= 7882
7. 2 and 4 and 6: n= 64
8. Remove duplicates from 7: n= 59

PubMed Advanced Search Builder < access the online database until December 2023, 31th>

Search: (propensity score) AND (ustekinumab) AND (vedolizumab)
("propensity score"[MeSH Terms] OR ("propensity"[All Fields] AND "score"[All Fields]) OR "propensity score"[All Fields]) AND ("ustekinumab"[MeSH Terms] OR "ustekinumab"[All Fields]) AND ("vedolizumab"[Supplementary Concept] OR "vedolizumab"[All Fields])
Translations
propensity score: "propensity score"[MeSH Terms] OR ("propensity"[All Fields] AND "score"[All Fields]) OR "propensity score"[All Fields]
ustekinumab: "ustekinumab"[MeSH Terms] OR "ustekinumab"[All Fields]
vedolizumab: "vedolizumab"[Supplementary Concept] OR "vedolizumab"[All Fields]

Supplementary Table S2. Studies included in meta-analysis

STUDY and year of pubblication	***Design***	***N° Patients***	*UST (n)*	*VDZ (n)*
Alric et al. 2020	Retrospective study	239	107	132
Biemans et al. 2020	Retrospective study	138	69	69
Gebeyehu et al. 2022	Retrospective study	121	40	81
Kappelman et al. 2022	Retrospective study	1368	884	484
Lenti et al. 2021	Retrospective study	393	275	118
Manlay et al. 2021	Retrospective study	312	224	88
Onali et al. 2022	Retrospective study	172	86	86
Singh et al. 2022	Retrospective study	442	221	221
Yang et al. 2023	Retrospective study	378	240	138
Garcia et al. 2024	Retrospective study from a prospective registry	835	628	207

Supplementary Table S3. PRISMA 2020 check list.

Section and Topic	Item #	Checklist item	Location where item is reported
**TITLE**			
Title	1	Identify the report as a systematic review.	Pag. 1
**ABSTRACT**			
Abstract	2	See the PRISMA 2020 for Abstracts checklist.	Pag. 2
**INTRODUCTION**			
Rationale	3	Describe the rationale for the review in the context of existing knowledge.	Pag. 1-2
Objectives	4	Provide an explicit statement of the objective(s) or question(s) the review addresses.	Pag. 2
**METHODS**			
Eligibility criteria	5	Specify the inclusion and exclusion criteria for the review and how studies were grouped for the syntheses.	Pag. 2
Information sources	6	Specify all databases, registers, websites, organisations, reference lists and other sources searched or consulted to identify studies. Specify the date when each source was last searched or consulted.	Pag. 2
Search strategy	7	Present the full search strategies for all databases, registers and websites, including any filters and limits used.	Pag. 2
Selection process	8	Specify the methods used to decide whether a study met the inclusion criteria of the review, including how many reviewers screened each record and each report retrieved, whether they worked independently, and if applicable, details of automation tools used in the process.	Pag. 2-3

Section and Topic	Item #	Checklist item	Location where item is reported
Data collection process	9	Specify the methods used to collect data from reports, including how many reviewers collected data from each report, whether they worked independently, any processes for obtaining or confirming data from study investigators, and if applicable, details of automation tools used in the process.	Pag. 2-3
Data items	10a	List and define all outcomes for which data were sought. Specify whether all results that were compatible with each outcome domain in each study were sought (e.g. for all measures, time points, analyses), and if not, the methods used to decide which results to collect.	Pag. 3
	10b	List and define all other variables for which data were sought (e.g. participant and intervention characteristics, funding sources). Describe any assumptions made about any missing or unclear information.	Pag. 3
Study risk of bias assessment	11	Specify the methods used to assess risk of bias in the included studies, including details of the tool(s) used, how many reviewers assessed each study and whether they worked independently, and if applicable, details of automation tools used in the process.	Pag. 3
Effect measures	12	Specify for each outcome the effect measure(s) (e.g. risk ratio, mean difference) used in the synthesis or presentation of results.	Pag. 2-3
Synthesis methods	13a	Describe the processes used to decide which studies were eligible for each synthesis (e.g. tabulating the study intervention characteristics and comparing against the planned groups for each synthesis (item #5)).	Pag. 2-3
	13b	Describe any methods required to prepare the data for presentation or synthesis, such as handling of missing summary statistics, or data conversions.	Pag. 2-3
	13c	Describe any methods used to tabulate or visually display results of individual studies and syntheses.	Pag. 2-3
	13d	Describe any methods used to synthesize results and provide a rationale for the choice(s). If meta-analysis was performed, describe the model(s), method(s) to identify the presence and extent of statistical heterogeneity, and software package(s) used.	Pag. 3
	13e	Describe any methods used to explore possible causes of heterogeneity among study results (e.g. subgroup analysis, meta-regression).	Pag. 2-3

Section and Topic	Item #	Checklist item	Location where item is reported
	13f	Describe any sensitivity analyses conducted to assess robustness of the synthesized results.	Pag. 2-3
Reporting bias assessment	14	Describe any methods used to assess risk of bias due to missing results in a synthesis (arising from reporting biases).	Pag. 3
Certainty assessment	15	Describe any methods used to assess certainty (or confidence) in the body of evidence for an outcome.	Pag. 2-3
**RESULTS**			
Study selection	16a	Describe the results of the search and selection process, from the number of records identified in the search to the number of studies included in the review, ideally using a flow diagram.	Pag. 3-4
	16b	Cite studies that might appear to meet the inclusion criteria, but which were excluded, and explain why they were excluded.	Pag. 3-5
Study characteristics	17	Cite each included study and present its characteristics.	Pag. 3-5
Risk of bias in studies	18	Present assessments of risk of bias for each included study.	Pag. 5
Results of individual studies	19	For all outcomes, present, for each study: (a) summary statistics for each group (where appropriate) and (b) an effect estimate and its precision (e.g. confidence/credible interval), ideally using structured tables or plots.	Pag.5-8
	20a	For each synthesis, briefly summarise the characteristics and risk of bias among contributing studies.	Pag. 5-8

Section and Topic	Item #	Checklist item	Location where item is reported
Results of syntheses	20b	Present results of all statistical syntheses conducted. If meta-analysis was done, present for each the summary estimate and its precision (e.g. confidence/credible interval) and measures of statistical heterogeneity. If comparing groups, describe the direction of the effect.	Pag. 5-8
	20c	Present results of all investigations of possible causes of heterogeneity among study results.	Pag. 4-8
	20d	Present results of all sensitivity analyses conducted to assess the robustness of the synthesized results.	Pag. 3-8
Reporting biases	21	Present assessments of risk of bias due to missing results (arising from reporting biases) for each synthesis assessed.	Pag. 5
Certainty of evidence	22	Present assessments of certainty (or confidence) in the body of evidence for each outcome assessed.	Pag. 5-8
**DISCUSSION**			
Discussion	23a	Provide a general interpretation of the results in the context of other evidence.	Pag. 8-9
	23b	Discuss any limitations of the evidence included in the review.	Pag. 9
	23c	Discuss any limitations of the review processes used.	Pag. 9
	23d	Discuss implications of the results for practice, policy, and future research.	Pag. 9
**OTHER INFORMATION**			
Registration and protocol	24a	Provide registration information for the review, including register name and registration number, or state that the review was not registered.	Pag. 3
	24b	Indicate where the review protocol can be accessed, or state that a protocol was not prepared.	Pag. 3

Section and Topic	Item #	Checklist item	Location where item is reported
	24c	Describe and explain any amendments to information provided at registration or in the protocol.	Pag. 3
Support	25	Describe sources of financial or non-financial support for the review, and the role of the funders or sponsors in the review.	Pag. 9
Competing interests	26	Declare any competing interests of review authors.	Pag. 9
Availability of data, code and other materials	27	Report which of the following are publicly available and where they can be found: template data collection forms; data extracted from included studies; data used for all analyses; analytic code; any other materials used in the review.	Pag. 9

From: Page MJ, McKenzie JE, Bossuyt PM, Boutron I, Hoffmann TC, Mulrow CD, et al. The PRISMA 2020 statement: an updated guideline for reporting systematic reviews. BMJ 2021;372:n71. doi: 10.1136/bmj.n71. This work is licensed under CC BY 4.0. To view a copy of this license, visit https://creativecommons.org/licenses/by/4.0/

Supplementary Table S4. Detailed bias risks report with authors' statements

Study and year of publication	Bias due to confounding	Bias in selection of participants	Bias in classification interventions	Deviation from intended intervention	Bias due to missing data	Measurement of outcomes	Selection of reported results
Onali, 2022	demographic data and disease characteristics after PSM were reported	Patients with incomplete data were excluded. Bias due to propensity score matching: PSM statement was addressed, model used and analysis to estimate PS were declared, baseline and weighted variables and sample size were described	none	none	After propensity score matching clinical remission at week 14; prior surgeries and drug discontinuation at 1 year are missing	Drug discontinuation rate was not considered at week 52 as weel as SF clinical remission at 14 weeks	results are reported properly
Singh, 2022	Smoking habit and disease characteristics after PMS in VDZ and UST cohort are missing	Patients were included if no new prescription of drugs were found in the system. Bias due to PMS: PSM statement was addressed, model used and analysis to estimate PS were declared, baseline and weighted variables and sample size were described	none	none	Clinical remission, drug discontinuation and adverse events are not reported	Abscesses were excluded from infection cause	results are reported properly
Lenti, 2021	Demographic data and disease characteristics after PSM were reported	Patients with incomplete data were excluded; in VDZ cohort were excluded patients anti-TNF naive and who was treated previously with UST (not specified in UST cohort). Bias due to PSM: PSM statement was addressed, model used and analysis to estimate PS were declared, baseline and weighted variables and sample size were described	none.	none	Safety outcomes (serious infection, adverse events and hospitalization in the first year of treatment) after PSM were not reported	Clinical remission at week 24 was not reported	Results are reported properly
Manlay, 2021	Demographic data and disease characteristics after PSM were reported	Patients treated with UST or VDZ in prevention of post-operative recurrence were excluded. Bias due to PSM: PSM statement was addressed, model used and analysis to estimate PS were declared, baseline and weighted variables and sample size were described	none	none	Serious infection and hospitalization in the first year of treatment were not reported	Drug discontinuation rate was not considered at week 52	Results are reported properly
Kappelman, 2022	Smoking habit and disease characteristics	Patients were selected using ICD-10 and National Drug Codes. Bias due to PSM: PSM statement was addressed, model used and analysis to estimate PS were	none	none	Clinical remission and adverse events were missing	Secondary analysis was made using a different cohort	Results are reported properly

	after PSM are missing	declared, baseline and weighted variables and sample size were described					
Gebeyehu, 2022	Disease duration and localization are missing after PMS	Selected only patients +60 years old, patients with insufficient data excluded. Bias due to PSM: PSM statement was addressed, model used and analysis to estimate PS were declared, baseline variables and sample size were described, but weighted variables were missed, only analysis results were available	none	none	Hospitalization at one year of treatment and adverse events were not reported	none	Results are reported properly
Alric, 2020	Demographic data and disease characteristics after PSM were reported	Bias due to PSM: PSM statement was addressed, model used and analysis to estimate PS were declared, baseline and weighted variables and sample size were described	none	none	none	none	Results are reported properly
Biemans, 2020	Demographic data and disease characteristics after PSM were reported	Bias due to PSM: PSM statement was addressed, model used and analysis to estimate PS were declared, baseline and weighted variables and sample size were described	none	none	All of the outcome of interest were reported	none	Results are reported properly
Yang, 2023	Demographic data and disease characteristics after PSM were reported	Bias due to PSM: PSM statement was addressed, model used and analysis to estimate PS were declared, baseline and weighted variables and sample size were described	none	none	Hospitalization at one year of treatment, 12-week clinical remission, drug discontinuation were not reported	none	Results are reported properly
Garcia, 2024	Demographic data and disease characteristics after PSM were reported	Bias due to PS: PS statement was addressed, model used and analysis to estimate PS were declared, but weighted variables were not clearly described	none	none	Hospitalization at one year of treatment were not reported	none	Results are reported properly
